# Recent Advances in Natural Materials for Corneal Tissue Engineering

**DOI:** 10.3390/bioengineering8110161

**Published:** 2021-10-26

**Authors:** Julie F. Jameson, Marisa O. Pacheco, Henry H. Nguyen, Edward A. Phelps, Whitney L. Stoppel

**Affiliations:** 1 Department of Chemical Engineering, University of Florida, Gainesville, FL 32611, USA; julie.jameson@ufl.edu (J.F.J.); marisa.pacheco@ufl.edu (M.O.P.); 2 Department of Materials Science and Engineering, University of Florida, Gainesville, FL 32611, USA; xhenryhnguyen@gmail.com; 3 J. Crayton Pruitt Family Department of Biomedical Engineering, University of Florida, Gainesville, FL 32611, USA; ephelps@bme.ufl.edu

**Keywords:** corneal tissue engineering, natural biomaterials, cornea, biomimetic materials

## Abstract

Given the incidence of corneal dysfunctions and diseases worldwide and the limited availability of healthy, human donors, investigators are working to generate engineered cellular and acellular therapeutic approaches as alternatives to corneal transplants from human cadavers. These engineered strategies aim to address existing complications with human corneal transplants, including graft rejection, infection, and complications resulting from surgical methodologies. The main goals of these research endeavors are to (1) determine ideal mechanical properties, (2) devise methodologies to improve the efficacy of engineered corneal grafts and cell-based therapies, and (3) optimize transplantation of engineered tissue structures in the eye. Thus, recent innovations have sought to address these challenges through both in vitro and in vivo studies. This review covers recent work aimed at evaluating engineered materials, potential therapeutic cells, and the resulting cell-material interactions that lead to optimal corneal graft properties. Furthermore, we discuss promising strategies in corneal tissue engineering techniques and in vivo studies in animal models.

## 1. Cornea Structure and Clinical Motivations for Engineered Therapies

Corneal injuries and defects resulting in vision loss affect over 1 million new patients worldwide each year [1]. Progressive diseases and injuries necessitate clinical repair or replacement of the cornea. Currently, the gold standard treatment for corneal disease or injury is the transplantation of allogenic materials that originate from human donor tissue [2]. Corneal surgery, or keratoplasty, for transplantation can be performed in two main ways, namely conventional penetrating keratoplasty or modern lamellar keratoplasty. There is a growing emphasis on the use of modern lamellar keratoplasty strategies, such as the Descemet’s membrane endothelial keratoplasty for posterior lamellar keratoplasty, or deep anterior lamellar keratoplasty for anterior lamellar keratoplasty, instead of conventional methods [3]. Human donor tissues have the appropriate physical and chemical signals and structure, and, when transplanted properly, partially alleviate symptoms of corneal injuries or disease in patients, often restoring some vision [4]. According to a 2016 global survey [2], over 185,000 corneal transplants are performed each year. This addresses only 1 in 70 patients who need a donor cornea worldwide [2]. In the United States, corneal transplants are performed approximately 40,000 times each year [5], making the cornea the most transplanted organ in the body [6]. Unfortunately, healthy, transplantable allografts (donor corneas) are of limited availability, failing to meet surgical demands and patient needs [2,7]. Additionally, cornea allografts are highly susceptible to graft rejection and failure due to elevated immune response over time, and where additional complications, such as vascularization, exist as function of the underlying causes necessitating the original transplant [3,4,8,9]. It has even been shown that other events that stimulate immune system activity, such as vaccination following the transplant [10,11], can have a negative impact on corneal transplant success, or require additional management of patient outcomes.

**Figure 1:**
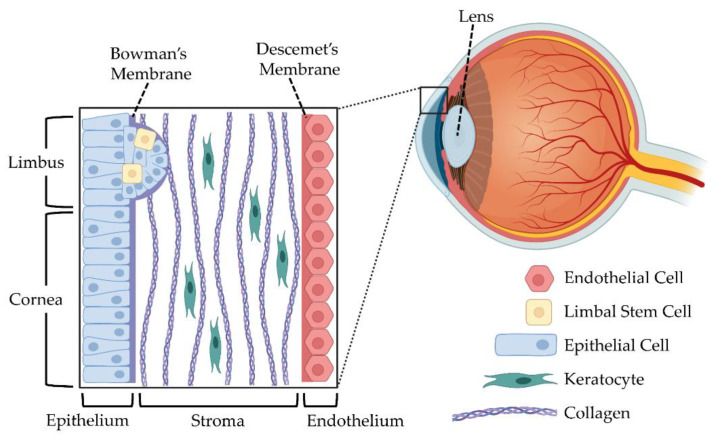
The human cornea constitutes the outermost layer of the eye. This transparent, avascular tissue consists of multiple layers including the epithelium, stroma, and endothelium. Other components include the limbus, the Bowman’s membrane, and the Descemet’s membrane. Figure created with Biorender.com.

To understand the ramifications of clinical procedures and develop new treatment modalities, it is critical to understand the biological structure of cornea tissue. The human cornea is an avascular and transparent connective tissue that forms a portion of the anterior segment of the eye [5,12], as shown in Figure 1.

The cornea consists of three main layers: epithelium, stroma, and endothelium. Each of these layers have specific functions, and tissue engineers have sought to develop mimics of the entire cornea, as well as independently replicate the specific roles and needs within each layer using a variety of cell types, as depicted in Table 1.

The epithelium comprises between five and seven layers of epithelial cells that regulate corneal hydration and act as a barrier between the environment and the eye [6,13]. Corneal epithelial stem cells, or limbal cells, have been thought to exist towards the basal layer of the limbus, near the epithelium, where the conjunctiva transitions to the cornea [14,15,16]. The stroma is dominated by highly organized collagen I and collagen V fibrils with quiescent keratocytes [17,18]. Lastly, the endothelium is the innermost layer of the cornea, and consists of a single layer of endothelial cells. The endothelium acts as a barrier and a functional pump for corneal clarity [19,20]. With corneal transparency being a major functional characteristic of the cornea, disorders of or injury to any of these layers can cause corneal opacity and visual disruption. Some of the most common progressive disorders causing visual disruption include epithelial disorders, such as limbal stem cell deficiency, stromal disorders, such as corneal dystrophy, and endothelial disorders, such as bullous keratopathy [13].

The shortage of allografts for repair of corneal damage may be addressed through engineering intelligent cornea biomimetics that mitigate symptoms of corneal disease, and restore some vision loss. This requires the development of engineered corneal constructs (cellularized) or scaffolds (acellular) with similar biochemical and structural characteristics to native corneal tissue that maintain optical clarity, and do not cause unnecessary scarring. Thus, corneal tissue engineering strategies currently focus on (1) matching the mechanical properties of native corneal tissue; (2) utilizing corneal cell types to achieve functionality; and (3) translating biomaterial research to clinical applications. When considering the development of corneal transplants, investigators often debate the use of all-natural or biologically derived biopolymers compared to the use of synthetic polymers. While recent advancements exist in the use of synthetic materials for corneal replacements [5,21,22], the scope of this review covers the use of natural biopolymers in the generation of transplantable tissues or in vitro cornea mimics. We emphasize recent work aimed at understanding mechanical properties of natural engineered materials, cell-material interactions, and biomaterial implantation in animal models in vivo.

## 2. Engineering a Cornea Mimetic: Mechanical Properties and Biomaterial Structure

To treat corneal injuries and diseases, the field of corneal tissue engineering has started to investigate natural materials as alternative treatment strategies for engineering the cornea, aiming to mitigate the shortcomings of allogenic corneal grafts [5,6,12]. Common natural materials investigated for these applications include silk fibroin, alginate, gelatin, collagen, chitosan, cellulose, hyaluronic acid (HA), and decellularized corneas. These natural materials have been synthetically improved and modified to expand the relevance for these applications, such as the methacrylation of gelatin to form GelMA, and the grafting of poly (vinyl alcohol) (PVA) onto the backbone of a natural biopolymer.

An important demand for functional engineered corneal constructs is the ability to create a construct that has similar mechanical properties to native cornea, so as to not induce undesirable responses from cells within the eye, such as stromal cells, alter the function of or put strain on eye muscles, or cause an unwanted immune response. The elastic modulus of the entire cornea ranges from 0.3–3.3 MPa [23], with variations in different layers, but also some discrepancies in measurements from different techniques. Additionally, the elastic modulus of the cornea is dependent on age [24], as the elastic modulus increases as patients age. The elastic modulus of the stroma is a function of depth within the stroma, with a higher reported elastic modulus higher in the anterior stroma (epithelial side) compared to the posterior stroma (endothelial side) [25]. However, the elastic modulus of the anterior stroma when measured by atomic force microscopy can range from 0.03–3 MPa [25,26,27]. Further, the Bowman’s membrane and Descemet’s membrane were found to have an elastic modulus of ~110 and ~50 kPa, respectively [26,28]. Disparities in technical measurements can result from many factors, including sample preparation, hydration of the tissue, and source of the tissue.

Furthermore, all corneal tissue layers are transparent with minimal light scattering, as assessed with light transmittance at varying wavelengths [29], key features for enabling vision. Thus, a major design consideration for engineered corneal transplants is the maintenance of transparency throughout the duration of the material’s use in vivo. Overall, when engineering mimics of natural tissues with eventual transplantation in mind, it is critical to consider the mechanical and physical properties of the tissue and their utility in vivo. Additionally, when designing engineered cornea mimics for use in the laboratory in order to understand the impact of pharmaceuticals on cell phenotypes, monitoring changes in mechanical and physical properties of the engineered tissue over time is critical for complete investigation.

### 2.1. Natural Biopolymer-Based Hydrogels as Cornea Mimics

Hydrogels are 3D networks of synthetic or natural polymers that can absorb a large amount of water. With the ability to construct hydrogels using different processing methods and different polymers, researchers can manufacture scaffolds with desired physiochemical and biomechanical properties. Recent advances in corneal tissue engineering have utilized hydrogel materials in innovative ways, and these are highlighted in Table 2. Recent studies, reported in Table 2, are organized first by the layer of the cornea that the researchers aimed to mimic, followed by the biomaterial used to construct the mimic. Table 2 emphasizes the methods used to assess the hydrogel system, as well as whether cells were used in the design or the assessments. Lastly, we identify any assessments in animal models to evaluate matters such as safety, efficacy, and function. Many combinations of polymers have been explored, and studies continue to show promise in developing a corneal tissue transplant [30,31,32,33,34]. In this section, we highlight of the recent advancements presented in Table 2, organized by the biopolymer used to form the hydrogel to enable a better direct comparison between studies.

#### 2.1.1. Silk Fibroin Hydrogels

Silk fibroin, or silk, is a natural biopolymer used in tissue engineering applications, often isolated from *Bombyx mori* silk cocoons. When isolated from silkworms, the silk fibroin protein is made of two large biopolymers, termed the heavy and light chains. Silk peptides, or small portions of the silk fibroin protein, can be genetically engineered and produced by *Escherichia coli* (*E. coli*) or other organisms in a bioreactor in a laboratory. These silk peptides often mimic sequences from spider silk, and can be coupled with other peptide sequences [35,36,37]. Regenerated silk fibroin protein [38] can be used to form hydrogels [39,40], or hydrogels can be formed from recombinantly produced peptides [41,42]. Regenerated silk fibroin can be modified to enhance functionality via modification of native or enriched carboxylic and amide groups on the protein backbone [43,44], and hydrogels can be formed through amino acid coupling reactions [39,45,46]. When engineered to mimic the cornea epithelium [47], silk-based hydrogels maintained transparency across a range of compressive moduli (0.4–11.5 kPa) by modulating the amount of metal ions present in the silk nanostructures [47]. When formulated to mimic the stroma, composite hydrogels formed from silk fibroin, genipin-crosslinked PVA, and nanocrystalline hydroxyapatite improved the structural integrity of the hydrogel network, compared to hydrogels without the permanent genipin crosslinking, while also maintaining human corneal fibroblast viability [48]. While transparent silk hydrogels can be produced, the slow formation of nanocrystalline domains within regenerated silk hydrogels can alter the transparency and mechanical properties of these materials [49], limiting the application of un-modified regenerated silk fibroin for cornea tissue engineering applications. Thus, ongoing work aims to prevent the formation of nanocrystalline domains or disrupt the phase separation that occurs during beta-sheet formation within these materials [46]. These modified regenerated silk fibroin-hyaluronic acid composite hydrogels have been used to form vitreous humor substitutes [50].

#### 2.1.2. Collagen-Based Hydrogels

As one of the major components of the extracellular matrix (ECM), type I collagen has been used as a natural hydrogel material for corneal substitution [51,52,53,54,55,56,57,58,59,60,61,62,63]. Crosslinking of type I collagen generates a collagen hydrogel, where the mechanical properties, pore size, and degradability of the system are controlled via the crosslinking method (physical, chemical, enzymatic) [62,64,65]. When utilizing type I collagen to generate epithelial mimics of cornea tissue, methacrylation and subsequent crosslinking of helical type I collagen biopolymers via Michael addition of thiols afforded a range of mechanical properties from pascals to kilopascals, while maintaining collagen biopolymer structure, maintaining proliferation of human corneal epithelial cells [52]. To mimic a stromal defect, a bio-orthogonally crosslinked hyaluronate-collagen in situ forming hydrogel [54] was evaluated for applications in corneal tissue engineering, given that the crosslinking reaction can occur under ambient conditions without the use of external light, photo-initiators, or heat. The resulting hyaluronate-collagen hydrogel had high transparency, quick gelation time, degradability, and cytocompatibility in vitro [54]. Nevertheless, these in situ forming hydrogels only achieved an elastic modulus up to 2 kPa for all formulations with differing collagen and hyaluronate concentrations [54], which is orders of magnitude lower than reported measurements of human stroma (0.03–3 MPa [25,26,27]).

Three-dimensional bioprinting has been used to advance the development of collagen cell-laden corneal tissue constructs that can match the biomechanics and geometry of native corneal tissue [51,53,55,58,66]. However, optical clarity, cell viability, and maintenance of cell phenotypes continue to pose challenges for these methodologies. Recent advancements using a drop-on-demand (DoD) bioprinting technique that can reduce shear stress at the printing extrusion nozzle led to a construct that mimicked the native human cornea stroma in geometry and shape, with a compressive tangent modulus of 18 kPa [55]. Another promising method of 3D bioprinting uses laser-assisted bioprinting (LaBP), generating a layered 3D bioprinted cornea epithelium [58]. While the full thickness cornea mimic was not transparent, the thickness and structure resembled that of the native human cornea [58]. Alternatively, pneumatic 3D extrusion bioprinting has been used to print collagen and alginate composite hydrogels to mimic the stroma, using anatomical features obtained from topographic data of an adult human cornea [53]. These advancements in 3D printing demonstrate the promise of this technique in the manipulation of type I collagen hydrogels, as engineers and scientists aim to maintain optical clarity and cell viability while balancing mechanical properties, such as elastic modulus, or in vivo activity, such as biodegradability.

**Table 2:** Summary of current advances in natural hydrogel formulations for corneal tissue engineering.

Natural Base Material (s)	Novelty	Research Model			Ref.
		Material Characterization	Cells for In Vitro Evaluation	In Vivo or Ex Vivo Studies	
**Epithelium**					
Silk fibroin	Production of transparent silk hydrogels with tunable mechanical properties using organic solvents and metal ions	–Mechanical properties –Morphology –Transparency	–Human CEpCs –Human dermal fibroblasts	--	[47]
Collagen/gelatin/alginate	Three-dimensional printing human CEpCs laden constructs with tunable degradation based on varying inclusion of sodium citrate	–Degradation –Morphology –Transparency	–Human CEpCs	--	[51]
Collagen	Methacrylation of collagen for improved flexibility	–Degradation –Mechanical properties –Spectroscopy	–Human CEpCs –Murine CPCs	--	[52]
Gelatin	Injectable, photocurable gelatin system, consisting of acrylated gelatin and thiolated gelatin, with tunable mechanical, biodegradation, and biological properties	–Degradation –Mechanical properties –Morphology –Spectroscopy –Transparency	–L929 murine fibroblasts	–Focal corneal injury in NZW rabbits	[67]
Chitosan	Thermosensitive chitosan-gelatin hydrogels releasing stromal cell derived factor-1 alpha	–Morphology	–Rat LESCs –Rat MSCs	–Alkali burn-injury in SD rats	[30]
**Stroma**					
Silk fibroin	PVA/silk/nano-hydroxyapatite hydrogels with structurally enhancing genipin crosslinking	–Degradation –Mechanical properties –Morphology	–Human CFs	--	[48]
Collagen	Three-dimensional printed collagen-I based bio-ink with varied amounts of methacrylated collagen and sodium alginate	–Mechanical properties –Morphology –Transparency	–Human CKs	--	[53]
Collagen	Bio-orthogonally crosslinked hyaluronate-collagen hydrogel	–Degradation –Mechanical properties –Morphology –Transparency	–Human CEpCs	–Anterior lamellar keratoplasty in NZW rabbits	[54]
Collagen	3D printable collagen/agarose	–Mechanical properties –Transparency	–Human CKs	--	[55]
Gelatin	Gelatin/ascorbic acid cryogels	–Degradation –Mechanical properties –Morphology –Spectroscopy	–Rabbit CKs	–Anterior lamellar keratoplasty in NZW rabbits	[68]
GelMA	Visible light cross-linkable	–Degradation –Mechanical properties	–Human CFs	–Half thickness stromal defect in NZW rabbits	[69]
GelMA	Three-dimensional printed using stereolithography	–Morphology –Transparency	–Human CSCs	--	[70]
GelMA	GelMA hydrogel with PCL-PEG scaffold support	–Mechanical properties –Transparency	–Rat LSSCs	–Intrastromal keratoplasty in SD Rats	[71]
GelMA	Hybrid Cellularized GelMA and decellularized bovine corneal ECM	–Degradation –Mechanical properties –Spectroscopy –Transparency	–Bovine CKs	--	[72]
GelMA	Three-dimensional printed with organized, encapsulated keratocytes	–Degradation –Mechanical properties –Morphology –Spectroscopy –Transparency	–Human CKs	--	[73]
Bacterial Cellulose	Composite Bacterial cellulose and PVA	–Morphology –Transparency	–Human CSCs	–Intrastromal keratoplasty in NZW rabbits	[31]
**Endothelium**					
GelMA	Nanopatterning and hybrid crosslinking for improved monolayers	–Degradation –Mechanical properties –Morphology –Spectroscopy	–Human CEnCs	–Anterior keratoplasty in NZW rabbits	[74]
HA	Porous HA hydrogel as endothelial cell sheet delivery system	–Degradation –Morphology	–Rabbit CEnCs	–Endothelial scrape wound in NZW rabbits	[32]
Poly-ε-lysine	Porous hydrogel for expansion of corneal endothelial cells with improved expansion when ECM proteins were adsorbed to the surface	–Mechanical properties –Transparency –Contact angle	–Human CEnCs (HCEC-12) –Porcine CEnCs		[21]
**Full Thickness**					
Collagen	Bottom-up assemblies of decellularized porcine corneal sheets and human CSC laden collagen gel layers with human CEpCs seeded at top surface	–Degradation –Transparency	–Human CSCs –Human CEpCs	–Ex vivo porcine anterior lamellar keratoplasty model	[57]
Collagen + Laminin	Laser assisted bioprinting with collagen and laminin-based inks	–Degradation –Morphology	–Human LESCs –Human ASCs	--	[58]
Collagen-like peptides	Collagen-like peptides/PEG/fibrinogen liquid hydrogel matrix that gels spontaneously at body temperature	–Mechanical properties –Transparency	–Human CEpCs	–Epithelial perforation wound in NZW rabbits –Anterior lamellar keratoplasty in Gottingen mini pigs	[59]
Collagen	Dual-layered collagen vitrigel with synthetic Bowman’s membrane and stromal layer. Contains ECM microparticles	–Degradation –Mechanical properties –Morphology –Transparency	–Rabbit CFs	–Anterior lamellar keratoplasty in NZW rabbits	[60]
Collagen	In situ forming collagen gels crosslinked with PEG-NHS have tunable transparency, degradation, and stiffness	–Degradation –Mechanical properties –Transparency	–Human CEpCs –Human LSSCs	–Keratectomy in NZW rabbits	[61]
Decellularized Porcine cornea	Thermoresponsive in situ forming hydrogel	–Mechanical properties –Morphology –Transparency	–Human CEpCs –Human MSCs	--	[33]

#### 2.1.3. Gelatin and GelMA Based Hydrogels

An alternative to helical type I collagen with secondary structure is the use of gelatin or methacrylated gelatin (GelMA) [12,56,67,68,69,71,72,73,74,75,76,77]. Gelatin, a denatured and hydrolyzed form of collagen, and its methacrylated form, have demonstrated broad applicability across many tissue engineering applications due biocompatibility, tailorable mechanical properties, low cost, ease of processing, and transparency [76,78], making them an attractive biomaterial for ocular applications. Gelatin cryogels (hydrogels formed under cold temperatures) with ascorbic acid incorporation provide tunable mechanical properties as a function of ascorbic acid concentration [68]. The elastic modulus decreased with the addition of ascorbic acid from 17.5–1.1 MPa, which more closely matches reported values of the stromal tissue. However, the inclusion of ascorbic acid to the gelatin cryogel increased the pore size and porosity of the cryogel, which was hypothesized to be due to interruption of the EDC crosslinking by the ascorbic acid [68].

Methacrylation of gelatin and subsequent photocrosslinking with a range of wavelengths of light, including visible light [69], and photoinitiators can yield greater control over crosslinking density and hydrogel porosity. The elastic modulus can be varied from 1.1–~220 kPa by changing the polymer concentration and light exposure time, or a combination of crosslinking strategies, including both physical crosslinking and UV crosslinking [74]. Use of both physical crosslinking and UV crosslinking demonstrated that the addition of a cooling step to the GelMA fabrication process facilitated additional crosslinking that had an 8-fold increase in mechanical strength compared to regular GelMA hydrogels [74]. Additional experiments verified that the mechanical stiffness of the GelMA did not decrease with an increase in temperature from 10–40 °C, and the rate of biodegradation for the hybrid crosslinked GelMA hydrogels was slower compared to that of only UV crosslinked GelMA hydrogels [74]. Nevertheless, this method of crosslinking showed that the rate of degradation can be tuned, an attractive attribute to match the healing cascade of the cornea injury or disease. Photocrosslinked GelMA can also be 3D printed to effectively mimic corneal stroma tissues [73], improving the structure and scalability of GelMA-based hydrogel stromal tissue mimics. Formation of GelMA:poly (2-hydroxyethyl methacrylate) (HEMA) composite hydrogels can also mimic corneal stroma tissues, facilitating greater control over mechanical properties and mechanical performance [77]. HEMA has a history of use in contact lenses and other ocular applications [79]. The addition of HEMA to the GelMA hydrogels increased the compressive modulus to a value of ~155 kPa, while still maintaining hydrogel transparency. Furthermore, the degradation experiments on GelMA:HEMA hydrogel when in the presence of collagenase type II solution showed that stability is maintained after 4 h at an enzyme concentration of 10 U/mL [77].

### 2.2. Films and Non-Hydrogel-Based Scaffolds as Cornea Mimics

Films and non-hydrogel-based scaffolds, such as electrospun mats, are emerging as promising mimics for corneal tissue engineering. Films have the capacity to preserve the properties of the bulk material, while tuning the surface features for improved cell responses. Furthermore, films can provide proper nutrient diffusion and, to advance cell-cell interactions, are appropriate for mimicking the structure of the human cornea. Non-hydrogel-based scaffolds can be fabricated without adding a crosslinking initiator, while still boosting the mechanical integrity through use of higher polymer concentrations. Current progress with natural biopolymer-based films and non-hydrogel-based scaffolds contribute to corneal tissue repair and regeneration (Table 3).

#### 2.2.1. Silk Fibroin Films

Silk fibroin, a natural protein extracted from *Bombyx mori* cocoons, can be formed into films and scaffolds with tunable degradation and mechanical strength with non-immunogenic responses in vivo [38,80,81]. Use of silk fibroin as a film or scaffolds for corneal tissue replacement [82,83,84,85,86,87,88,89,90,91,92,93,94,95,96,97,98,99,100] remains an active area of research, as outlined in Table 3. Silk films and membranes can be fabricated through simple evaporation or electrospinning. Silk films prepared with differing methanol concentrations, a technique to induce β-sheet crystallinity within the secondary structure of the silk polymer, resulted in elastic modulus values ranging from 13–445 kPa [82]. Furthermore, a centrifugal casting technique has been developed to improve the mechanical properties of silk films [83]. The elastic modulus was increased from 31–43 MPa when centrifugal casting was employed to create the silk films over dry casting. The centrifugal cast film also resulted in decreased surface roughness and enhanced optical transparency [83].

The addition of polyurethane to an electrospun silk film reduced the elastic modulus from 5–0.1 MPa [84]. The addition of poly (ε-caprolactone) (PCL) also decreased the elastic modulus of lyophilized electrospun silk scaffolds from 2.6–1.2 MPa [85]. Electrospinning aligned silk scaffolds improved the transparency to levels near the native human cornea. Gelatin and GelMA can be incorporated into silk films and scaffolds to improve cell material interactions [86,87,88]. A scaffold with 60 weight % silk and 40 weight % gelatin had a tensile strength of 27 MPa. This biocompatible scaffold was able to stabilize and deliver glial cell line-derived neurotrophic factor (GDNF), which contributed to enhanced healing in an epithelium-stroma defect [88]. Silk films with 30% GelMA by volume had the highest elastic modulus at 440 kPa, over GelMA and silk on its own [87]. Additional silk films with a 30% GelMA core and an ascorbic acid loaded alginate film layer had a markedly higher elastic modulus of 660 kPa, compared to 40 kPa when the film was only composed of silk and GelMA [86].

Silk proteins sourced from silkworms other than *Bombyx mori* cocoons are also being researched for their potential in corneal tissue engineering. Silk proteins isolated from *Philosamia ricini* (PR) and *Antheraea assamensis* (AA) silkworms can be fabricated into silk films by casting [101]. Films prepared from AA had a 1.5 higher fold change in the elastic modulus compared to *Bombyx mori* and PR silk films, while maintaining similar level of transparency.

**Table 3:** Summary of current advances in natural scaffold and film engineering for corneal tissue.

Materials	Novelty	Research Model			Ref
		Material Characterization	In Vitro Cell Studies	In Vivo or Ex Vivo Studies	
**Epithelium**					
Silk fibroin	Optimization of silk fibroin, poly-D-lysine coated silk fibroin, RGD modified silk fibroin, and poly-D-lysine blended silk fibroin films for human corneal epithelium growth	–Spectroscopy	–Human CEpCs	--	[102]
Silk fibroin	Silk films with nanotopography and extracellular proteins	–Morphology	–Murine CEpCs –Rabbit CEpCs	–Corneal epithelium debridement in C57BL/6 mice	[89]
Silk fibroin	Silk film with tunable stiffness and cellular effects	–Mechanical properties –Morphology	–Human CEpCs	--	[82]
Silk fibroin	Various silk film surface features of various pitch and width dimensions ranging from the micro- to nanoscale	–Morphology	–Human CEpCs	--	[103]
Silk fibroin	Hybrid silk/PU electrospun mat for corneal epithelial differentiation of conjunctiva-derived MSC	–Mechanical properties –Morphology	–Human MSCs	--	[84]
Silk fibroin	Fabrication and biocompatibility of electroconductive silk/PEDOT/PSS composites	–Degradation –Mechanical properties –Spectroscopy –Transparency	–Human CEpCs	--	[90]
Silk fibroin	PEG modified silk membranes as a carrier for limbal epithelial stem cells transplantation	–Morphology	–Rabbit LESCs	–Limbal stem cell deficiency NZW rabbit model	[91]
Collagen	Collagen/chondroitin sulfate film with high moisture capacity	–Mechanical properties –Spectroscopy –Transparency	–Human CEpCs	--	[104]
Collagen	Collagen film with micro-rough surface	–Morphology	--	–Lamellar keratoplasty in NZW rabbits	[105]
Chitosan/gelatin/HA	Carboxymethyl chitosan/gelatin/HA membrane as transplantation scaffold for corneal wound healing	–Transparency	–Rabbit CEpCs –Rabbit CSCs	–Alkali burn-injury in NZW rabbits	[106]
**Stroma**					
Silk fibroin	Contact guidance by RGD-treated silk films stacked in an orthogonally, multi-layered architecture to control the alignment and distribution of human LSSCs	–Mechanical properties –Morphology –Spectroscopy –Transparency	–Human LSSCs –Human CFs	--	[92]
Silk fibroin	Investigate the in vivo response and the effect of silk crystalline structure on degradation rates of silk films in rabbit multipocket corneal models	–Spectroscopy	--	–Corneal multipocket model in NZW rabbits	[93]
Silk fibroin	Silk film using centrifugal casting technique for corneal tissue engineering	–Mechanical properties –Morphology –Transparency	–Human CKs	--	[83]
Silk fibroin	Influence of surface topography and mechanical strain on keratocyte phenotype and ECM formation	–Morphology –Transparency	–Human CKs	--	[94]
Silk fibroin	Multi-lamellar human corneal stroma tissue in vitro by differentiating periodontal ligament stem cells towards keratocytes on an aligned silk membrane	–Morphology	–Human periodontal ligament stem cells	--	[95]
Silk fibroin	Corneal stromal regeneration by hybrid silk/PCL electrospun scaffold	–Degradation –Mechanical properties –Morphology –Spectroscopy –Transparency	–Human CKs	--	[85]
Silk/ GelMA	Transparent hybrid silk/GelMA films for cornea tissue engineering	–Degradation –Mechanical properties –Morphology –Spectroscopy –Transparency	–Human CFs	--	[87]
Silk/ GelMA	Double-layer film with ascorbic acid reservoir sodium alginate adhesive and anisotropic layer of micro-patterned silk nanofibrils incorporated with gelatin methacrylate for stroma tissue engineering	–Degradation –Mechanical properties –Morphology –Spectroscopy –Transparency	–Human CSCs	--	[86]
Collagen	Examine the influence of compositional and structural differences on keratocyte behavior	–Degradation –Morphology –Transparency	–Bovine CKs	--	[107]
Collagen	Pure collagen-based biomimetic 3D corneal stromal model constructed from pure electro-compacted collagen	–Degradation –Mechanical Properties –Morphology –Transparency	–Human CSCs	--	[108]
Decellularized bovine corneal matrix	Examine the influence of compositional and structural differences on keratocyte behavior	–Degradation –Morphology –Transparency	–Bovine CKs	--	[107]
Decellularized human stromal refractive lenticules	Femtosecond laser-derived human stromal lenticules decellularized with sodium dodecyl sulfate could produce transplantable biomaterial	–Morphology –Transparency	–Human CFs	–SMILE surgery in NZW rabbits	[109]
**Endothelium**					
Silk fibroin	Silk-based artificial endothelial graft for use in a rabbit Descemet’s membrane endothelial keratoplasty	–Mechanical Properties –Transparency	–Human CEnCs –Rabbit CEnCs	–Descemet membrane endothelial keratoplasty surgery in NZW rabbits	[96]
Silk fibroin	Transparent ultrathin film scaffolds with nature-derived aloe vera gel and silk	–Morphology –Spectroscopy –Transparency	–Rabbit CEnCs	–Descemet’s stripping and endothelial keratoplasty in NZW rabbits	[97]
Silk fibroin	Silk/β-Carotene films for delivery of corneal endothelial cells to replace diseased corneal endothelial cells	–Morphology –Spectroscopy –Transparency	–Rabbit CEnCs	--	[98]
Silk fibroin	Silk/lysophosphatidic acid films as a substrate for corneal endothelial cell delivery	–Morphology –Spectroscopy	–Rabbit CEnCs	--	[99]
Silk fibroin	Transparent silk/glycerol film, as a potential substrate for corneal endothelial cell regeneration	–Morphology –Spectroscopy –Transparency	–Rabbit CEnCs	--	[100]
Philosamia ricini silk	Non-mulberry silk for the culture of corneal endothelium	–Degradation –Mechanical properties –Morphology –Spectroscopy –Transparency	–Human CEnCs	--	[101]
Antheraea assamensis silk	Non-mulberry silk for the culture of corneal endothelium	–Degradation –Mechanical properties –Morphology –Spectroscopy –Transparency	–Human CEnCs	--	[101]
Collagen	Collagen/PLGA as a substrate for corneal endothelial cell regeneration	–Degradation –Morphology –Transparency	–Rabbit CEnCs	--	[110]
**Full Thickness**					
Silk fibroin	Thin silk protein film stacks as the scaffolding to support the corneal epithelial and stromal layers, and a surrounding silk porous sponge to support neuronal growth	--	–Human LSSC –Human CEpCs –Chicken dorsal root ganglion cells	--	[111]
Silk fibroin	Combining the corneal stroma and epithelium into one co-culture system, to monitor both human LSSC and human CEpC growth and differentiation into keratocytes and differentiated epithelium	--	–Human LSSCs –Human CEpCs	--	[112]
Silk fibroin	Biodegradable silk fibroin-based scaffolds containing glial cell line-derived neurotrophic factor for re-epithelialization	–Degradation –Mechanical properties –Morphology	–Human CKs	–Epithelial-stromal damage in C57BL/6 J mice	[88]
Collagen	Microgroove films as an external cue for cell responses	–Degradation –Transparency	–Rabbit CEpCs –Rabbit CKs	--	[113]
Collagen	Incorporation of cellulose nanocrystals into collagen films for improved mechanical properties	–Degradation –Mechanical properties –Morphology –Transparency	–Rabbit CEpCs –Rabbit CKs	--	[114]
Collagen	Collagen/PVAc nanofibrous electrospun scaffold suitable for cornea tissue engineering	–Mechanical properties –Morphology –Transparency	–Human CKs –Human CEpCs	--	[115]
Collagen	3D hemispherical transparent scaffold with radially aligned nanofibers fabricated with the designed peg-top collector	–Mechanical properties –Morphology –Spectroscopy –Transparency	–Rabbit corneal cells	--	[116]
Decellularized porcine cornea	Construct a full-thickness artificial cornea substitute in vitro by coculturing limbal epithelial cell-like cells and corneal endothelial cell-like cells derived from human embryonic stem cells on scaffolds	–Mechanical properties –Transparency	–Human CEpCs –Human CFs –Human embryonic stem cells	–Penetrating keratoplasty in NZW rabbits	[117]
Decellularized porcine cornea	Method using supercritical carbon dioxide to prepare acellular porcine cornea	–Mechanical properties –Morphology	--	–Anterior lamellar keratoplasty in NZW rabbits	[118]
Decellularized porcine corneal scaffolds	Decellularized corneas by formic acid, acetic acid, and citric acid treatment for corneal regeneration	–Mechanical properties –Transparency	–Human CEpCs –Rabbit CKs	–Deep anterior lamellar keratoplasty in NZW rabbits	[119]

#### 2.2.2. Collagen Films and Electrospun Mats

Promising materials, such as collagen, have admirable characteristics regarding cytocompatibility, biodegradability, and ease of production, but lack the mechanical toughness and elasticity needed for clinical applications. As mentioned above, the corneal stroma naturally contains collagen I and V, making collagen a clear choice for use in corneal biomaterials [17,18]. Furthermore, tripeptide arginine-glycine-aspartic acid (RGD) is a repeating motif on collagen, and critical for cell adhesion, migration, and proliferation. Collagen has thus been studied as films and scaffolds for cornea mimetics [105,107,108,110,114,115,116] as outlined in Table 3.

Electrochemical compaction is a technique utilized during scaffold fabrication to increase the mechanical stability of the material [120]. Chen et al. used electrochemical compaction on pure collagen I, and observed a 5-fold increase in the elastic modulus when compared to noncompacted collagen. When stromal cells were introduced to the material, the transparency decreased slightly from 88–82%, which is still suitable for use in the cornea [108].

Synthetic polymers are often incorporated into collagen-based mimetics to increase the mechanical properties needed for clinical applications [121]. Aligned polyvinyl acetate (PVAc) and collagen scaffold was electrospun, and improved the tensile strength to 3.6 MPa [115]. However, the addition of PVAc to the scaffold decreased the transmittance at wavelengths to between 400–650 nm. PCL was also used as a supplement in an electrospun collagen scaffold to form a hemispherical radially aligned cornea mimitic [116]. The radially aligned scaffold had an elastic modulus of 11 MPa, and acceptable transparency for implantable cornea mimetic [116].

To improve the mostiure capacity of collagen films, chondroitin sulfate can be added. Chondroitin sulfate is a sulfated glycosaminoglycan, and an important structural component of ECM [122]. Its polyanion groups have a high water retention capacity, making it a candidate to improve moisture retention in cornea films and scaffolds [123]. The elastic modulus decreased from 18.8–18 MPa with the addition of chondroitin sulfate, but there was no difference in the transparency, reaching a maximum of 85% [104]. However, the incorporation of cellulose nanocrystals increased the elastic modulus of collagen film from 0.67–1.82 MPa when the concentration of cellulose nanocrystals was increased to 10 wt% [114]. The transparency decreased as the concentration of cellulose nanocrystals increased due to the phase separation between the two polymeric systems [114].

#### 2.2.3. Decellularized Corneal Tissues

Decellularized corneas have similar mechanical properties and 3D structure to native human corneas; however, they can illicit an immune response or cause rejection if residual genetic or cellular material is present. Porcine and bovine corneas are often used as the source material, since human corneas are in short supply, while corneas can be easily obtained from agricultural industry. Researchers have made strides in understanding how to effectively decellularize tissue and engineer effective cornea mimics [107,109,117,118,119,124,125].

Since replicating the ECM components and architecture of native tissue has been a challenge, using decellularized scaffolds has great potential. A full thickness cornea substitute, comprised of decellularized porcine cornea seeded with limbal epithelial-like cells and corneal fibroblasts (CFs), maintained transparency while having an elastic modulus of 0.05 GPa [117]. However, decellularized corneas washed in triton and treated with supercritical CO_2_ had decreased elastic modulus, compared to native porcine cornea [118]. The gross transparency of the supercritical CO_2_-treated decellularized corneas matched that of the native porcine cornea. Other methods for decellularizing corneas use acetic acid, formic acid, and citric acid [119]. All forms of acid treatment decreased the elastic modulus, with citric acid decreasing the elastic modulus by 2-fold. The transmittance from citric acid-treated corneas was slightly reduced, but not statistically different. Acetic acid- and formic acid-treated corneas had comparable transmittance to the native porcine cornea.

## 3. Engineering a Cornea Mimetic: Cell Types and Their Function

Corneal tissue engineering techniques have advanced through research of cells, to address weaknesses of biomaterial integration and short-term viability. As seen previously, the development of corneal tissue substitutes often integrates cells into the materials to improve tissue-implant interactions. Alternative uses for these cells include the production of cell sheets, or the direct application of cells to the surface of the eye or inside the eye via injection. For certain diseases, such as limbal stem cell deficiencies, cell replacement or augmentation therapy may suffice when a full cornea replacement is not necessary. These applications have been recently reviewed [126,127,128,129,130,131], and are outside the scope of this manuscript. Here, we will specifically emphasize the development of cornea mimics that use cells in combination with natural biomaterials that explore patient outcomes in animal models or serve as in vitro tissue models. Many cell types have been investigated for use in corneal tissue engineering, including corneal endothelial cells (CEnCs), corneal epithelial cells (CEpCs), corneal epithelial stem cells (CEpSCs), keratocytes (CKs), mesenchymal stem cells (MSCs), adipose-tissue derived stem cells (ASCs), and embryonic-derived stem cells [132,133,134].

### 3.1. Cells for Corneal Epithelium

The corneal epithelium is the outermost layer of the cornea. Its main function is to protect the eye from pathogen penetration, provide light refraction through integration of tears, and act as a barrier for fluid flow [6,13,135,136]. It is comprised of five to seven layers of epithelial cells, with a cobblestone like morphology (see Figure 1 and Table 1). Adjacent cells are held together by desmosomes, while the basal cells are held to the basal lamina by hemidesmosomes and filaments [137,138]. The limbus contains epithelial stem cells that maintain epithelium homeostasis, as these cells are mitotically active [139,140].

Researchers commonly use human corneal epithelial cells for biomaterial experimentation [30,51,89,90,102,103]. These cells can either be from an immortalized cell line, or freshly isolated from donor epithelium. Epithelial cells can be identified by the expression of markers such as cytokeratin 3 (CK3), cytokeratin 12 (CK12), cytokeratin 15 (CK15), involucrin, tumor protein 63 (p63), aldehyde dehydrogenase 3 family, member A1 (ALDH3A1), and gap junction protein connexin-43 (Cx43) (Table 1). Cytokeratins are found in the cytoplasmic cytoskeleton, and are a component of intermediate filaments. Specifically, CK3 and CK12 can form strong intermediate filaments that assemble into strong networks that provide resistance to mechanical stress. Involucrin is a transglutaminase substrate protein, and a differentiation marker for corneal epithelial cells. p63 is a transcription factor of the p53 gene family, and plays a role in the proliferative capacity of corneal epithelial cells. ALDH3A1 is an enzyme that comprises 10–40% of the soluble proteins in the cornea. It is thought to protect the cornea from oxidative damage by UV and 4-hydroxy-2-nonenal. Cx43 is a component of gap junctions which allows cells to directly connect the cytoplasm of cells.

Nanopatterned materials are also used to match the topographical features of the corneal basement membrane. These physical cues can help to direct and guide cell adherence, migration, and proliferation. Researchers are exploring the use of nanopatterning with silk fibroin to accelerate corneal wound healing. One study investigated the cytoskeletal architecture and cellular gene expression of primary isolated human CEpCs, and immortalized human CEpCs seeded on silk films with varying surface features [103]. Actin filament alignment, as stained with Phalloidin, occurred along the silk film grooves and the vinculin co-localized with the actin filaments. They also showed that after 72 h of culture, the cells exposed to nanopatterned silk surfaces in comparison to flat silk surfaces expressed higher gene expression of paxillin, vinculin, and integrin β-1. However, by day 14, there were no changes in the expression of paxillin and integrin β-1. They hypothesize that actin re-organization continues to mediate downstream signaling over time, while the expression of integrin β-1 may be decreased after initial cell attachment. Another group created nanopatterned silk films with coatings of either collagen I, fibronectin, laminin, or poly-D-lysine coatings [89]. Adherence of primary mouse and rabbit CEpC number increased as the ridge width decreased on noncoated nanopatterned silk films. Cells prefer to align and migrate parallel to the topography. They also found that silk film coatings of collagen I significantly increased cell numbers, in comparison to fibronectin and laminin coatings and have synergistic effects with nanotopography to enhance cell growth.

ECM molecule addition to natural biomaterials can improve cell migration and proliferation. Jia et al. created arginine-glycine-aspartic acid (RGD)-modified silk films and poly-D-lysine coated silk films for epithelium regeneration [102]. Poly-D-lysine coated silk films promoted human CEpC adhesion at early phases in comparison to RGD modified silk films. The combination of modifying the silk film with RGD and coating the silk film with poly-D-lysine promoted the most cell migration in a scratch assay. They also confirmed that the addition of RGD and poly-D-lysine improved viability and proliferation of human CEpCs. Recently, Wu et al. printed an alginate/gelatin gel with collagen I and human CEpCs [51]. This alginate/gelatin/collagen I gel with human CEpCs allowed for a stable material that achieved high cell viability (94.6 ± 2.5%). In addition, the sodium citrate induced degradation aided in the proliferation of the human CEpCs and synthesis of CK3 over 5 days.

Alternative sources for stem cells in biomaterials have also been researched for corneal tissue engineering, such as dental pulp derived cells [141]. Dental pulp, a vascularized connective tissue, is found in the center of the tooth. Stem cells in the dental pulp have been characterized as multipotent, and are able to differentiate into cells, such as the corneal epithelium, which presents potential for corneal repair [142]. Also, unlike other stem cell populations, such as bone and adipose, dental pulp stem cells share developmental origins with the corneal stroma, which could be a potential advantage [127]. One study delivered dental pulp stem cells using contact lenses onto the corneal surface, which resulted in enhanced repair and regeneration of the human corneal epithelium [141]. This source of stem cells has translational potential, since they can be easily accessed and isolated.

### 3.2. Cells for Corneal Stroma

The corneal stroma is the thickest layer of the cornea, and comprises most of the cornea [18,135]. It sits between the endothelial layer and the epithelial layer. The stroma consists of highly aligned collagen I and V fibrils, which give the stroma its transparency and mechanical properties. Within the stroma, CKs (highly specialized quiescent stromal cells) regulate the production of ECM proteins and proteoglycans [143,144].

Studies use many corneal stromal cell sources for investigating cell-material interactions in stromal tissue engineering strategies, including human CKs, bovine CKs, human CSCs, human LSSCs, and rat LSSCs [31,70,71,72,75,92,94,107]. The use of biomarkers to identify stromal cells include keratocan, lumican, α-actinin, aldehyde dehydrogenase 1 family, member A1 (ALDH1A1), and aldehyde dehydrogenase 3 family, member A1 (ALDH3A1). Keratocan and lumican are keratan sulfate proteoglycans important for corneal transparency. α-actinin is a microfilament imperative for the attachment and binding of actin filaments. ALDH1A1 is a water-soluble enzyme that helps to maintain the corneal transparency. The stroma is also often characterized by the presence of collagen I through immunohistochemistry.

Human CKs were investigated within a 3D corneal model, fabricated with silk fibroin [78]. A silk film with surface topography was treated with a dome-shaped mechanical strain to resemble the shape of the native cornea. A silk film created with 600 mm^−1^ and 3% mechanical strain enhanced gene expression of lumican, keratocan, collagen I, and collagen V, in comparison to nonpatterned and unstrained silk films. Bektas et al. produced photopolymerizable GelMA hydrogels seeded with human CKs that mimic the human corneal stroma [75]. The human CKs had ~90% viability on both day 1 and day 2. However, after 21 days in culture, human CKs near the edge of the scaffold created networks with elongated phenotypes, while cells deep within the hydrogel were more circular in shape, reflecting insufficient nutrient diffusion to the center of the scaffold [75]. However, immunohistochemical staining of collagen I and V showed expression within the hydrogel [75].

Human CSCs are another cell studied in biomaterial platforms. One study used a visible light-based stereolithography 3D bioprinting method with GelMA and encapsulated CSCs [70]. The CSCs within the GelMA hydrogels had significantly higher gene expression of collagen I, lumican, and keratan sulfate at day 7 and day 14, as compared to CSKs cultured on tissue culture plates. Lastly, a bacterial cellulose/PVA hydrogel was created as a tissue engineered corneal stroma [31]. Human CSCs seeded on the bacterial cellulose/PVA hydrogels showed an increase in gene expression of ALDH3, lumican, and keratocan, compared to CSCs cultured on bacterial cellulose substrates alone [31].

A major type of cell used in corneal tissue engineering is human LSSCs. LSSCs have been popular and utilized in cell engineering for their broad differentiation potential, and ability to modulate the immune response [145]. In one study, nanopatterned silk films modified with RGD were stacked in an orthogonally, multi-layered architecture to allow for a 3D culture system for LSSCs [92]. After 9 weeks in culture, human LSSCs had an up-regulation of genes for keratocan, lumican, and ALDH3A1, as compared to day 0. Histological hematoxylin and eosin staining also showed endogenous ECM production over 9 weeks [92].

### 3.3. Cells for Corneal Endothelium

The human corneal endothelium consists of a layer of hexagonally closed packed CEnCs, with an average cell density around 3000 cells/mm^2^ [146,147,148]. Once the layer is formed, the CEnCs become mitotically inactive [149]. As the patient ages, the cell count decreases, while the surface area increases to compensate. The endothelial cornea acts as a barrier for hydration, making sure the stroma maintains a water content of ~78% [150]. It also acts as a pump to maintain transparency. As the cell density drops, the endothelium loses its ability to perform these functions, which is why the major requirement for endothelial tissue replacement is cell count. Biomaterials containing CEnCs are being studied as ways to recover function and cell density, while avoiding full tissue replacement [32,74,99,100].

Recently, researchers have used both rabbit and human CEnCs in studies. These cells are typically isolated from donor eyes, and then maintained in culture before being introduced to a biomaterial platform. Functional markers for these cells include a tight junction marker zonula occludens (ZO-1) and Na+/K+ ATPase, which is involved in the pump functions of the endothelial layer (Table 1). In one study, the concentration of HA hydrogels was varied to find ideal conditions for culture and delivery of a rabbit endothelial cell sheet [32]. After 8 h, viability assays revealed that a medium concentration of HA (0.25 wt%) led to the most viable cells due to the porous structure. However, it was found that the low concentration hydrogel (0.05 wt%) led to the highest expression of genes associated with the Na+/K+ ATPase pump.

Other studies combined rabbit CEnCs with silk fibroin based films. Choi et al. explored how a composite lysophosphatidic acid/silk fibroin film guided cell behavior [99]. They found that the composite film had higher initial attatchment and proliferation over 7 days than the silk-only film. The composite films also had increased ZO-1 and Na+/K+ ATPase expression, as determined by immunofluoresence staining. Another group surface modified silk fims to include glycerol [100]. Results showed that attatchment and proliferation of rabbit CEnCs were improved with the inclusion of glycerol, but ZO-1 and Na+/K+ ATPase remained similar for films with and without glycerol [100].

Nanopatterning is another method of enhanching cell material interactions. Rizwan et al. created a novel GelMA based platform that was uniquely crosslinked, first physically and then by UV, and nanopatterned with pillers and wells at a high resolution [74]. While both patterened and unpatterned gels led to the formation of human CEnC monolayers with polygonal shapes, unpatterened gels had the lowest amount of ZO-1 and Na+/K+ ATPase expression, key markers of functional cells [74]. Additionally, patterened gels led to improved cell density and homogeneity [74], which are necessary for translation of a cellularized biomaterial into a clinical product.

### 3.4. Combining Cell Types for Partial and Full Thickness Mimics

To have a fully functional cornea mimic, cell types from each tissue layer must either be included in or recruited to the material. Recent works have utilized CSCs in combination with CEpCs to create cellularized systems that more completely represent the entire cornea, and perform all its necessary functions [57,58,111,112,117]. These systems have made use of primary isolated CEpCs, immortalized CEpCs, CSCs, and embryonic and adipose stem cells. Utilizing techniques such as RT-PCR and immunohistochemistry, groups looked for key markers expressed by CEpCs (Cx43, CK3, ALDH3A1, involucrin), CSCs and surrounding extracellular matrix (keratocan, lumican, collagen I), and endothelial cells (ZO-1, Na+/K+ ATPase) to assess the functionality of their constructs (Table 1).

To engineer a full-thickness cornea substitute, Zhang et al. utilized human epithelial and endothelial-like cells derived from embryonic stem cells in an acellular porcine corneal scaffold [117]. The initial choice of embryonic stem cells is advantageous, since human embryonic stem cells are derived from the inner cell mass of a blastocyst and display pluripotency, which can be preserved during long-term culture [151]. Zhang and others have previously published on the differentiation ability of embryonic stem cells to corneal-like cells [152,153]. Zhang et al. used cell sorting to take *N*-cadherin positive cells to seed on the endothelial portion of their construct, two weeks prior to taking ABCG2 positive cells to seed on the epithelial portion of their construct. They found the epithelial-like sorted cells to have the desired cobblestone like morphology and high expression of CK3, while the endothelial-like sorted cells were found to have polygonal morphology and high expression of ZO-1 and Na+/K+ ATPase.

Another group introduced human CSCs in a collagen hydrogel to decellularized porcine cornea, using a bottom-up strategy, by placing the cell-laden hydrogel in between acellular sheets and then seeding human immortalized epithelial cells onto the top surface of the construct two weeks later [57]. After 21 days in culture the stromal segment of the construct showed upregulated gene expression of keratocan, lumican, ALDH3A1, and collagen I. The epithelial-like surface also showed tightly packed cells with a cobblestone morphology.

Sorkio et al. also aimed to create a structure containing both stroma-like and epithelial-like segments, by using collagen and laminin based bioprinting [58]. Like Zhang, embryonic stem cell-derived cells were utilized in the epithelial layer; however, in the stromal section, ASCs were used. The epithelial segment was printed in laminin, while the stromal segment was printed in collagen and alternate between cellular and acellular sections. Before combining the two structures, they were analyzed separately and both were found to have high cell viability, and immunofluorescent staining demonstrated proliferation, with an increase in proliferation marker Ki67 for 4–7 days. After 12 days, CK 3 was observed in the printed epithelium-like structure. After 7 days, the stromal-like structure was found to have collagen organization like that of native stroma.

Other groups have also aimed at creating constructs with both epithelial and stromal segments [111,112]. Co-culture of LSSCs and CEpCs in a stacked silk fibroin film construct is a method to achieve this partial thickness cornea mimic. Gosselin et al. analyzed human LSSCs and human CEpCs cultured on 3D silk constructs alone and in co-culture [112]. Using immunohistochemical staining, they found that CEpCs in co-culture expressed more Cx43 and involucrin, and the LSSCs in co-culture expressed less of the stem cell marker BCRP. This indicates more complete differentiation to stromal-like cells and formation of tight junctions in the epithelial layer. They also used qPCR, and found that the co-cultured system led to increased mRNA expression of CK3 in the CEpCs and of ALDH3A1 in LSSCs. Wang et al. constructed a similar system, and introduced innervation [111]. To do so, LSSCs, CEpCs, and chicken dorsal root ganglion were all co-cultured on 3D patterned silk film stacks surrounded by a collagen hydrogel. The co-culture led to increased involucrin mRNA expression in CEpCs than under single culture conditions. Additionally, LSSCs expressed the most keratocan expression in a liquid co-culture than when in an air-liquid interface-based culture. Future goals are to include an endothelial layer and evaluate these constructs in vivo.

## 4. Investigations of Natural Biopolymer-Based Engineered Corneas in Pre-Clinical Models

Treatments for corneal disease include direct delivery of cells, implant of an allograft, or implant or delivery of an engineered tissue mimic. The scope of this section will cover recent advancements in generation and evaluation of engineered tissue mimics, and the components of these systems. Other strategies, such as cell injection or methods for improving allograft survival, have recently been reviewed elsewhere [126,154,155,156].

### 4.1. Silk Fibroin and Its Use in Pre-Clinical Models

Pre-clinical models to evaluate silk fibroin-based materials in vivo have been established [88,91,93,96,97]. Silk films seeded with and without rabbit CEnCs were transplanted into a NZW rabbit Descemet’s membrane endothelial keratoplasty model [96]. The addition of rabbit CEnCs to the silk film improved transparency over 6 weeks, as compared to the acellular silk film. Moreover, the cellularized film was attached tightly to the corneal stroma, and the rabbit CEnCs showed similar phenotypic expression of ZO-1 and Na+/K+ ATPase, as compared to the healthy control [96].

To enhance cell growth, researchers have incorporated other biomaterials into silk scaffolds for in vivo corneal tissue mimetics [91,97]. One group has incorporated silk with nature-derived aloe vera, for human CEnCs to produce a transparent ultrathin film scaffold [97]. In a NZW rabbit model of Descemet’s stripping and endothelial keratoplasty, both silk and aloe vera displayed tissue integration with positive staining of ZO-1 and the Na+/K+ ATPase. Other biomaterials, such as PEG, have been incorporated into silk membranes [91]. After removal of the limbus and corneal epithelium, what the authors call a “total limbal stem cell deficiency” model, cellularized PEG modified silk membranes were transplanted onto NZR rabbit corneas. Four months after transplantation, CK12 positive staining was observed, displaying a stratified epithelial layer. There was no evidence of scarring, epithelial conjunctivalization, corneal neovascularization, or inflammation. The transplanted LESCs survived and repopulated the limbus. Lastly, gelatin and glial cell line-derived neurotrophic factor (GDNF) was incorporated into silk films [88]. GDNF operates to trigger signal cascades, such as MAPK, ERK1/2, and JNK1/2, that stimulate proliferation, differentiation, and apoptosis [157,158,159]. Specifically, GDNF has been shown to prevent apoptosis. In an experimental C57BL/6J mouse model of epithelial-stromal damage, the addition of GDNF led to faster corneal epithelization, compared to scaffolds without GDNF. It was also determined that GDNF can stimulate epithelial cells and keratocytes through the MAP-kinase pathway via immunohistological staining of phospho-ERK1/2 and phospho-JNK1/2. Ki67 positive staining also confirmed the proliferative capacity of the epithelial cells and keratocytes.

### 4.2. Collagen-Based Materials in Pre-Clinical Models

Transparent collagen films, gels, and scaffolds have been investigated in pre-clinical models [54,59,60,61,105]. Liu et al. engineered a collagen film with a micro-rough surface to repair the epithelium [105]. Using a corneal lamellar keratoplasty model in NZW rabbits, they found that their material was suturable, and promoted complete reepithelization within 2 weeks. Moreover, ocular transparency was recovered and maintained after a month, while no signs of rejection of neovascularization were observed. A hyaluronate-collagen hydrogel was made to mimic and promote healing of the stromal and epithelial layers [54]. In a lamellar keratectomy model in rabbits, they were able to make a suture-less repair, in which, after one week, normal transparency and corneal curvature was observed. On day 7, ZO-1 was observed in the epithelial layer, and 5–8 layers of epithelial cells were observed in a healthy phenotype. Additionally, alpha smooth muscle actin was only very slightly expressed in the stromal segment, indicating keratocytes were not activated to a myofibroblast state associated with corneal scarring [105]. Another group also built collagen hydrogels for stromal and epithelial repair, and used poly (ethylene glycol) for crosslinking [61]. The gels were implanted in rabbits following a manual keratectomy. After one week, they observed transparency and epithelial migration. Rabbits that received the hydrogel treatment also had higher levels CK3 and ZO-1 expression, and lower alpha smooth muscle actin expression. Together, this is indicative of a promising regenerative material [61].

Cellularized collagen materials were developed to engineer both the Bowman’s membrane and a stromal layer. To address the variation in structure and cell type, a dual-layered collagen-based corneal substitute was developed [60]. The Bowman’s membrane component was created from a previously reported beta cyclodextrin (βCD)-mediated collagen vitrigel [160,161], while the stromal layer was a homogenous layer of collagen [60]. Moreover, porcine small intestinal submucosa (SIS) ECM microparticles were added to the stromal layer. When placed in a rabbit anterior lamellar keratoplasty model, the construct was suturable, and promoted full reepithelization within 2 weeks. Integration of the vitrigel with the host stroma was observed by day 30. Moreover, by day 30, multilayered epithelial cells showed expression of CK3 and ZO-1, while the stromal segment saw little to no expression of alpha smooth muscle actin [60]. Results are promising for the future of these materials for clinical translation.

### 4.3. Gelatin and GelMA Materials in Pre-Clinical Investigations

The use of gelatin-based materials in pre-clinical models is well-established [67,68,69,71,74,106,162], yet challenges exist in producing a mechanically similar material, and finding a suitable long-term in vivo model has been a bottleneck in recent developments. To increase the mechanical stability of gelatin hydrogels, Li et al. utilized thiol-ene chemistry to prepare acrylated gelatin and thiolated gelatin that could be crosslinked via a photoinitiated thiol-acrylate reaction [67]. In a NZW rabbit model of focal corneal injury, the use of the acellular gelatin hydrogel led to reepithelization in less than 3 days, as compared to the sham group, which took between 3–7 days. Observed by optical coherence tomography (OCT), the acellular gelatin hydrogel showed thinner and smoother scar tissue compared to the sham group. Another group made a fiber-reinforced GelMA hydrogel to try to improve the mechanical stability of GelMA hydrogels [71]. This allowed the load to be transferred to the fibers from the hydrogel. Orthogonally aligned PCL-PEG sub-microfibers were perfused with GelMA to form a 3D construct that allowed regeneration of the stroma in a rat intrastromal keratoplasty model in vivo. The transplanted hydrogels also showed high transparency over 3 months [71], a critical component for successful application to human patients.

The inclusion of cells into the biomaterial cornea mimetic has also shown favorable results in vivo. Transplanting cellularized gelatin hydrogels in NZW rabbit alkali burn-induced models restored corneal transparency and improved wound healing [68,106]. With the addition of ascorbic acid to the hydrogel, collagen type I deposition was observed, leading to corneal stromal matrix regeneration. The ascorbic acid incorporation also led to decreased alkali burn-induced oxidative damage [68]. When composite gelatin was fabricated with carboxymethyl chitosan and HA, it reduced the injury area, and increases in K3 and K12 in the epithelium and vimentin in the stroma were observed [106].

### 4.4. Decellularized Corneas

Corneal mimics made from decellularized components have demonstrated excellent biocompatibility and regeneration potential in vivo [109,117,118,119]. Most approaches using decellularized tissue implant acellular materials in the hopes of recruiting the correct cells to the injured sight. Hin-Fai Yam et al. utilized decellularized human stromal lenticules to make a stroma-like structure. This was, in turn, used in an in vivo study, where the scaffolds were placed into corneal stromal pockets on adult NZW rabbits [109]. Shortly after implantation, some opacity was observed; however, transparency was recovered after two weeks. Additionally, histology revealed lenticule integration with host tissue and an absence of immune cells after 21 weeks (approximately 5 months) [109]. TEM showed that the orientation of collagen fibrils in the lenticules had a less defined lattice structure than those in the host tissue [109].

Other groups have had success in using decellularized scaffolds as partial and full thickness cornea constructs. Where these types of materials often differ is in the method of decellularization. In vivo experiments can reveal immune responses and/or rejection in models where adequate decellularization is not achieved [163,164]. Lin et al. prepared acellular porcine scaffolds using organic acid treatment [119]. This treatment strategy resulted in up to a 99% decrease in DNA content within the scaffold, while only slightly decreasing the collagen content. The most successful group of decellularized scaffolds were then implanted into adult NZW rabbits’ deep anterior lamellar keratoplasty model. After week one, scaffolds remained transparent for the 60-day course of the experiment. Additionally, fluorescein staining showed reepithelization over the injured site. H&E staining also showed scaffold–host integration within the stromal layer, and no signs of rejection or unwanted vascularization occurred within the course of the experiment. Huang et al. prepared an acellular porcine cornea scaffold using a supercritical carbon dioxide-based approach [118]. This technique achieved a 75% decrease in DNA content, complete removal of whole cells, and allowed only collagen to remain. When placed in a lamellar keratoplasty model in adult NZW rabbits, no complications, neovascularization, or infections occurred over 6 months. Transparency was reached after two weeks, and was maintained throughout the rest of the experiment. H&E images at two months showed keratocytes entering the scaffold. At both two months and six months, immunohistochemistry showed CK3 and CK12 in the epithelial layer indicating successful reepithelization [118].

Zhang et al. achieved promising in vivo performance using a unique approach of recellularizing an acellular porcine cornea scaffold [117]. They used cell sorting to take *N*-cadherin positive embryonic-derived stem cells to seed on the endothelial portion of their construct, two weeks prior to taking ABCG2 positive embryonic-derived stem cells to seed on the epithelial portion of their construct. This final construct was implanted in a lamellar keratoplasty model in adult NZW rabbits. Following transplantation, they observed initial opacity that became transparent after two weeks, and remained transparent for two months. Moreover, over those two months, no signs of rejection or unwanted neovascularization were observed. Additionally, it was found that, after two months, the construct had successfully formed three to five layers of tightly packed epithelial like cells (positive for CK3), and a monolayer of tightly packed endothelial-like cells (positive for ZO-1 and Na+/K+ ATPase) [117]. Overall, pre-clinical models suggest that formulations of cells and natural biopolymers have potential to solve pressing problems in the treatment of diseased or damaged corneas.

## 5. Outlook

Until alternatives to human allografts are engineered for keratoplasty, there will remain a shortage of materials for repairing corneal defects or injuries in human patients. While recent advances in cellularized materials have led to better tissue mimics of the layers of the cornea, these efforts have not translated to new cellularized or acellular natural biopolymer implants reaching the market. Clinical trials over the last decade have explored the injection of several types of stem cell in low concentration biopolymer solutions [165,166], limited biomaterial systems [167,168], and xenogeneic materials [169], but cohorts of patients in these studies are small, compared to efforts to understand what leads to successful human allograft implantation and limits graft rejection. For example, a randomized clinical trial aimed to study the safety and feasibility of an engineered fibrin-agarose cornea scaffold containing HCEpCs, keratocytes, and growth factors [167,170]. Their preliminary results have indicated that the scaffolds can be safely implanted, with at least partial efficacy to reconstruct the corneal surface, and ongoing Phase I/II clinical trials focus on treating corneal ulcers [171]. Future work toward engineered alternatives for cornea for transplantation will require a scale-up of materials using good manufacturing practices (GMP) for the materials investigated, coupled with what can be a lengthy process of testing and approvals. Use of a natural material, such as collagen, gelatin, or silk fibroin, is advantageous, as there are already pathways for approval of these biopolymers for use in vivo, which may accelerate the path to translation over a newly generated synthetic material. Often, natural materials can be formulated in aqueous environments, reducing the potential for residual chemicals or pre-cursors in the final product. However, bridging the gap between material formulations and engineered products will require substantial collaboration [172] between clinical ophthalmologists and immunologists, to ensure that the developed and formulated products have lasting positive impacts on patient health. Often, engineered products can require very specific surgical procedures and techniques that are hard to replicate globally, as specific surgical training for use of a new material is not always accessible. While robotic arms and other surgical technique options are available at research hospitals, these are difficult to equitably translate across the globe.

Alternatively, ongoing work to generate tissue mimics for in vitro analysis of cell responses to pharmaceuticals or therapeutics is making great strides. Bioreactors enabling air liquid interface culture, as well as multilayer mimics [111,173], allow for exploration of hypotheses related to cell-cell interaction, protein production and paracrine signaling. Overall, innovations and advances in recent years highlight the complexity in translating exciting and promising technology from the laboratory to the clinic. Ongoing efforts must address persistent limitations, such as rejection and unwanted immune responses, and suboptimal mechanical properties.

## Figures and Tables

**Table 1:** Cell types used in corneal tissue engineering.

Cell Type	Abbreviation	Healthy Phenotype	Common Markers
Corneal Epithelial Cells	CEpCs	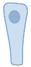 Cobblestone Close Packed	CK3 CK12 CK15 ALDH3A1 Cx43 Involucrin p63
Corneal Stromal Cells	CSCs	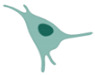 Dendritic	Keratocan Lumican *α*-actinin *α*SMA ALDH1A1 ALDH3A1
Corneal Endothelial Cells	CEnCs	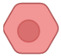 Hexagonally Close Packed	ZO-1 Na+/K+ ATPase
Adipose-tissue Derived Stem Cells	ASCs	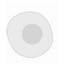	Ki67 p63 p40 CK3
Mesenchymal Stem Cells	MSCs	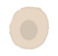	CD13 CD29 CK3 CK8 CK12
Limbal Epithelial Stem Cells or Corneal Epithelial Stem Cells	LESCs or CEpSCs	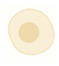	Ki67 p63 p40 CK3 ABCG2 CK19 EFGR Integrin β1

## Nomenclature

**Materials**	
ECM	Extracellular matrix
GelMA	Methacrylated gelatin
HA	hyaluronic acid
HEMA	poly (2-hydroxyethyl methacrylate)
PCL	poly-ε-caprolactone
PEG	polyethylene glycol
PVA	polyvinyl alcohol
PVAc	polyvinyl acetate
**Cell Types**	
ASCs	Adipose-derived stem cells
CEnCs	Corneal endothelial cells
CEpCs	Corneal epithelial cells
CFs	Corneal fibroblasts
CKs	Corneal keratocytes
CPCs	Cardiac progenitor cells
CSCs	Corneal stromal cells
LESCs	Limbal epithelial stem cells
LSSCs	Limbal stromal stem cells
MSCs	Mesenchymal stem cells
**Markers**	
ABCG2	ATP Binding Cassette family member G2
ALDH1A1	Aldehyde Dehydrogenase 1 family member A1
ALDH3A1	Aldehyde Dehydrogenase 3 family member A1
CD13	Cluster of Differentiation group 13
CD29	Cluster of Differentiation group 29
CK3	Cytokeratin-3
CK8	Cytokeratin-8
CK12	Cytokeratin-12
CK15	Cytokeratin-15
CK19	Cytokeratin-19
Cx43	Connexin-43
EFGR	Epidermal Growth Factor Receptor
p63	Tumor Protein 63
ZO-1	Zonula Occludens-1
*α* SMA	alpha smooth muscle actin
**Miscellaneous**	
EDC	*N*-(3-Dimethylaminopropyl)-*N*′-ethylcarbodiamide hydrochloride
LaBP	Laser assisted bioprinting
NHS	*N*-hydroxysuccinimide
NZW	New Zealand White
RGD	arginine-glycine-aspartic acid
SD	Sprague Dawley
